# Association Between Eosinophilic Esophagitis and Coded Ocular Diagnoses: A Retrospective Cohort Study

**DOI:** 10.3390/life16071156

**Published:** 2026-07-13

**Authors:** Yun-Feng Li, Yu-Jung Su, Hui-Chin Chang, Tien-Yun Lee, Meng-Che Wu, Shuo-Yan Gau

**Affiliations:** 1 School of Medicine, Chung Shan Medical University, Taichung 402302, Taiwan; 2 Orthopedics Department, Chi-Mei Medical Center, Tainan 710004, Taiwan; 3 Evidence-Based Medicine Center, Chung Shan Medical University Hospital, Taichung 402302, Taiwan; 4 Library, Chung Shan Medical University Hospital, Taichung 402302, Taiwan; 5 Department of Medical Education, Ditmanson Medical Foundation Chia-Yi Christian Hospital, Chiayi City 600566, Taiwan; 6 Division of Pediatric Gastroenterology, Children’s Medical Center, Taichung Veterans General Hospital, Taichung 40705, Taiwan; 7 Department of Post-Baccalaureate Medicine, College of Medicine, National Chung Hsing University, Taichung 402202, Taiwan; 8 Department and Graduate Institute of Business Administration, National Taiwan University, Taipei 106319, Taiwan; 9 Institute of Allergology, Charité—Universitätsmedizin Berlin, Corporate Member of Freie Universität Berlin and Humboldt-Universität zu Berlin, Berlin, Germany

**Keywords:** eosinophilic esophagitis, coded ocular diagnoses, real-world study

## Abstract

**Background:** Eosinophilic esophagitis (EoE) is a chronic immune-mediated disease that is increasingly recognized as a systemic inflammatory condition. Its potential association with subsequent coded ocular diagnoses has not been well characterized in large-scale longitudinal studies. **Methods:** We conducted a retrospective cohort study using the TriNetX Global Collaborative Network, which aggregates de-identified electronic health records from multiple international healthcare systems. Adults aged ≥18 years with at least two clinical encounters between 2005 and 2024 were included. Patients with EoE (ICD-10-CM K20.0) were identified as the exposure cohort, while individuals undergoing routine health examinations without EoE served as controls. Those with prior ocular disease, malignancy, or death were excluded. Propensity score matching (1:1) was used to balance demographics, body mass index, comorbidities, medication use, and socioeconomic factors. The primary outcomes were coded ocular diagnostic categories identified using ICD-10-CM codes. To reduce the likelihood of including pre-existing conditions, ocular disease events diagnosed within 3 months after the index date were excluded from the analysis. Hazard ratios (HRs) and 95% confidence intervals (CIs) were estimated. Sensitivity analyses incorporated alternative exposure definitions, washout periods, and follow-up durations, with additional stratification by age, sex, and race. **Results:** After matching, 64,613 patients were included in each cohort. EoE diagnostic coding was associated with a higher subsequent occurrence of several coded ocular diagnostic categories, including visual disturbance and blindness (HR = 1.521; 95% CI: 1.383–1.673), disorders of refraction and accommodation (HR = 1.324; 95% CI: 1.188–1.474), lacrimal system disorders (HR = 1.504; 95% CI: 1.274–1.775), cataract (HR = 1.637; 95% CI: 1.384–1.935), glaucoma (HR = 1.463; 95% CI: 1.157–1.849), and disorders of the vitreous body and globe (HR = 1.903; 95% CI: 1.510–2.399). These findings should be interpreted cautiously because several outcomes, such as visual disturbance, disorders of refraction and accommodation, and ocular pain, were broad diagnostic categories and may be susceptible to detection or coding practices. **Conclusions:** In this large-scale EHR-based cohort study, EoE diagnostic coding was associated with a higher subsequent occurrence of several coded ocular diagnostic categories. These findings should be interpreted as exploratory associations rather than evidence of direct causal or mechanistic relationships, particularly for broad or detection-prone outcomes such as visual disturbance, disorders of refraction and accommodation, and ocular pain.

## 1. Introduction

Eosinophilic esophagitis (EoE) is a chronic inflammatory disease that causes dysphagia, food impaction, esophageal stricture, and heartburn in adults, as well as abdominal pain, failure to thrive, and vomiting in children [1]. The pathogenesis of EoE is multifactorial, involving genetic, environmental, and immune factors, as well as food and inhaled protein antigens, which can induce a T helper type 2 (Th2) cell-mediated response. Interleukin (IL)-5 and IL-13 trigger eosinophil proliferation and activation, leading to subepithelial fibrosis and esophageal stenosis [2]. The diagnosis of EoE includes characteristic symptoms, histologic confirmation of >15 eosinophils/hpf on biopsy, and classic endoscopic findings [3]. Common medications, including proton pump inhibitors and topical corticosteroids, as well as monoclonal antibodies such as dupilumab and dietary elimination therapy, have been established as effective treatment options for patients with EoE [4].

EoE has been associated with numerous comorbidities. Previous studies have demonstrated strong associations between EoE and allergic manifestations, such as severe pediatric asthma, atopic dermatitis, allergic rhinitis, and pollen food allergy syndrome [5,6,7]. In a U.S. observational study, metabolic dysfunction-associated steatotic liver disease (MASLD) showed an increased incidence in the EoE group, with 2.38-fold higher odds, while inflammatory bowel disease, celiac disease, gastroesophageal reflux disease, and irritable bowel syndrome were also found to be associated with EoE [8]. In terms of ocular conditions, related atopic diseases such as vernal keratoconjunctivitis have been reported to be associated with EoE [9]. A potentially plausible clinical connection between EoE and ocular diagnostic categories may arise from the broader atopic and immune-mediated profile of EoE. EoE frequently coexists with allergic diseases, including asthma, allergic rhinitis, atopic dermatitis, and food allergy [1], suggesting that patients with EoE may represent a population with heightened atopic susceptibility and immune dysregulation. Some ocular conditions, particularly ocular surface or allergy-related diagnoses, may also be influenced by atopic inflammation and systemic immune activity [10]. Additionally, hidradenitis suppurativa (HS), another chronic inflammatory disease that shares similar immune mechanisms with EoE, has been shown to be associated with ocular diseases such as visual disturbances, disorders of refraction and accommodation, lacrimal system disorders, glaucoma, and blepharitis [11]. These observations provide the clinical rationale for examining whether EoE diagnostic coding is associated with a higher subsequent burden of coded ocular diagnostic categories.

Despite increasing recognition of EoE as an immune-mediated and atopy-associated disease, its relationship with the subsequent ocular diagnostic burden remains poorly characterized. Existing evidence has mainly focused on isolated allergic ocular conditions or ocular comorbidities in other chronic inflammatory diseases rather than on the longitudinal ocular diagnostic profile of patients with EoE. Therefore, a large-scale real-world cohort study may provide useful exploratory evidence to determine whether EoE diagnostic coding is associated with subsequent ocular diagnostic categories and to generate hypotheses for future clinically validated studies. Accordingly, we conducted a large-scale EHR-based cohort study using the TriNetX Global Collaborative Network to evaluate whether EoE diagnostic coding was associated with subsequent coded ocular diagnostic categories in a multicenter real-world setting.

## 2. Materials and Methods

This investigation was designed as a retrospective cohort analysis conducted across multiple centers, drawing upon data from the TriNetX research network. TriNetX is an internationally integrated electronic health record database that is continuously updated prospectively. It aggregates anonymized patient information contributed by participating healthcare institutions, making it a valuable platform for large-scale epidemiologic studies [12,13]. For the purposes of this research, data were specifically obtained from the Global Collaborative Network within TriNetX. Detailed information regarding the coding strategy is provided in Supplementary Table S1.

**Figure 1:**
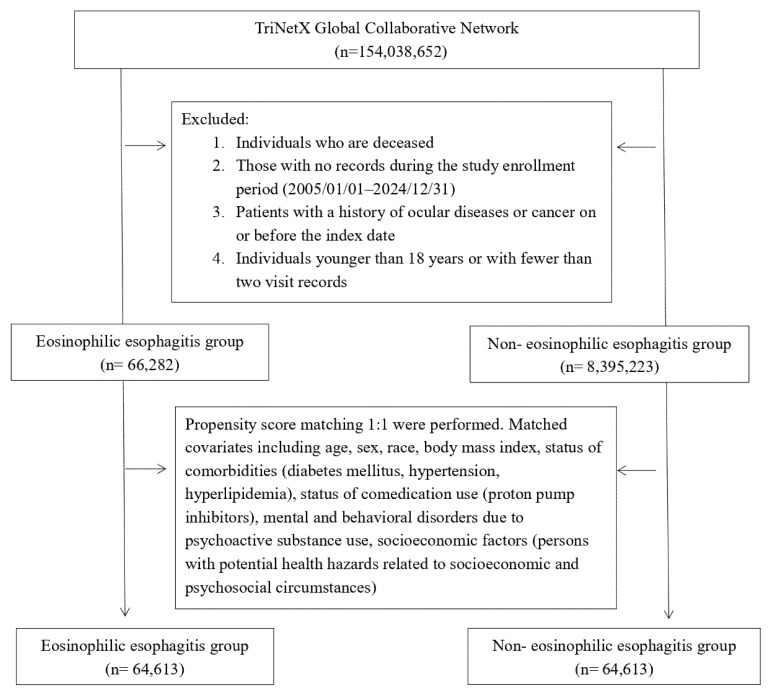
Patient selection flowchart.

Eligible individuals were adults aged 18 years or older who had at least two documented visits to medical institutions between January 2005 and December 2024 (see Figure 1). Patients diagnosed with EoE (ICD-10-CM K20.0) were assigned to the exposure group, although endoscopic and histological confirmation could not be conducted within our database network. On the other hand, individuals with records of encounters for adult health examination (ICD-10-CM Z00.0) without any prior EoE diagnosis served as the comparison group. Exclusion criteria applied to both groups included a death record, any history of malignancy, or documented ocular conditions (ICD-10-CM: H00–H59) before study entry.

To ensure comparability between groups, propensity score matching was implemented for each analysis. Baseline characteristics were evaluated both before and after matching to confirm balance. Variables included in the matching process comprised demographic factors (age, sex, race), body mass index, comorbid conditions (diabetes mellitus, hypertension, hyperlipidemia), medication exposure (proton pump inhibitors), substance-related mental and behavioral disorders, and indicators of socioeconomic vulnerability. Because the TriNetX analytics platform imposes a system-defined limit on the number of covariates that can be included in a single propensity score model, not all clinically relevant atopic or inflammatory comorbidities could be incorporated into the primary matching model. Therefore, additional exclusion-based sensitivity analyses were performed for asthma, allergic rhinitis, and noninfective enteritis and colitis to evaluate the potential influence of these residual imbalances.

The primary outcomes were predefined coded ocular diagnostic categories, including visual disturbance and blindness (ICD-10-CM H53–H54), disorders of refraction and accommodation (ICD-10-CM H52), disorders of the lacrimal system (ICD-10-CM H04), hordeolum of the eyelid (ICD-10-CM H00.0), blepharitis (ICD-10-CM H01.0), ocular pain (ICD-10-CM H57.1), cataract (ICD-10-CM H25, H26, H28), disorders of the sclera (ICD-10-CM H15), glaucoma (ICD-10-CM H40–H42), and disorders of the vitreous body and globe (ICD-10-CM H43–H44). The outcome categories were selected to comprehensively evaluate the spectrum of ophthalmologic comorbidities potentially associated with EoE in a real-world clinical setting. Similar ICD-10-CM-based outcome definitions have also been adopted in previous large-scale TriNetX studies investigating ocular diseases as comorbidities of chronic disorders [11]. However, these broad ICD-10-CM-based categories were used to characterize an ocular comorbidity profile in an exploratory EHR-based setting and should not be interpreted as specialist-confirmed inflammatory ocular diseases. To reduce potential bias, any outcomes occurring within 3 months following the index date were excluded. Additional analyses were conducted to test the robustness of the findings. These included multiple sensitivity analyses using alternative modeling strategies to limit overmatching effects. Furthermore, varying washout periods were incorporated to address the possibility of reverse causation.

All statistical analyses were performed using the built-in analytic functions of the TriNetX platform. Propensity score matching was performed at a 1:1 ratio using the TriNetX nearest-neighbor matching algorithm with a caliper width of 0.1. Matching was performed separately for each analysis. Baseline balance before and after matching was assessed using standardized mean differences (SMDs), with values greater than 0.1 considered indicative of meaningful imbalance. The detailed covariate sets used in the sensitivity analyses are summarized in Supplementary Table S2. Associations between EoE diagnostic coding and subsequent coded ocular diagnostic categories were estimated using the outcome comparison analytic function within TriNetX and are presented as hazard ratios (HRs) with 95% confidence intervals (CIs). Patients were followed from the index date until the first occurrence of the outcome of interest, the end of the available follow-up period, or the end of the study period, whichever occurred first. Ocular diagnoses recorded within 3 months after the index date were excluded to reduce the likelihood of capturing pre-existing ocular conditions.

Some statistical implementation details are platform-defined within TriNetX. The platform does not provide formal proportional hazards diagnostics, such as Schoenfeld residual testing, or detailed person-time incidence rates for each analytic model. Therefore, incidence rates per person-year and formal assessment of proportional hazards assumptions could not be reported. In addition, because this study evaluated multiple coded ocular diagnostic categories across several sensitivity and subgroup analyses, the analyses were considered exploratory. No formal correction for multiple comparisons was applied. Therefore, nominal *p*-values and 95% confidence intervals should be interpreted cautiously.

## 3. Results

### 3.1. Baseline Characteristics of the Study Participants

The baseline characteristics of the study participants before and after propensity score matching in both the EoE cohort and the control cohort are presented in Table 1. Before matching, significant differences were observed in variables including age, sex, race, comedication and comorbidities between the two groups with SMDs exceeding 0.1. After matching, the EoE cohort included 64,613 patients, and the non-EoE control group had the same number of participants (Figure 1). The mean age was 29.9 ± 16.5 years, and female participants accounted for 37.4% in both matched cohorts. The racial distribution was also similar between the two cohorts after matching, with 81.6% of patients being White and 4.7% being Black or African American. After matching, most demographic variables, body mass index, proton pump inhibitor use, systemic corticosteroid use, and several cardiometabolic comorbidities were well balanced between the two cohorts. However, residual imbalance remained for asthma, allergic rhinitis, and non-infective enteritis and colitis, with post-matching SMDs greater than 0.1. These residual differences were considered clinically relevant and were further evaluated using exclusion-based sensitivity analyses.

### 3.2. Association Between EoE Diagnostic Coding and Subsequent Coded Ocular Diagnoses

**Figure 2:**
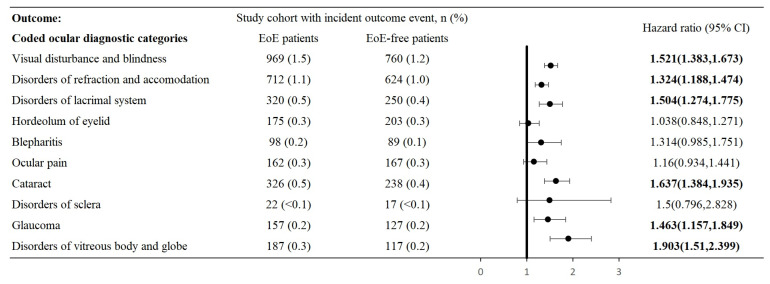
Risk of coded ocular diagnostic categories in EoE patients.

The association between EoE diagnostic coding and subsequent coded ocular diagnostic categories is presented in Figure 2. Compared with the control cohort during the 15-year follow-up period, patients with EoE diagnostic coding had a higher subsequent occurrence of several coded ocular diagnostic categories, including visual disturbance and blindness (HR: 1.521, 95% CI: 1.383–1.673), disorders of refraction and accommodation (HR: 1.324, 95% CI: 1.188–1.474), disorders of the lacrimal system (HR: 1.504, 95% CI: 1.274–1.775), cataract (HR: 1.637, 95% CI: 1.384–1.935), glaucoma (HR: 1.463, 95% CI: 1.157–1.849), and disorders of the vitreous body and globe (HR: 1.903, 95% CI: 1.510–2.399). Nevertheless, some ocular diseases did not show significantly increased risks in the EoE group, including hordeolum of the eyelid (HR: 1.038, 95% CI: 0.848–1.271), blepharitis (HR: 1.314, 95% CI: 0.985–1.751), ocular pain (HR: 1.160, 95% CI: 0.934–1.441), and disorders of the sclera (HR: 1.500, 95% CI: 0.796–2.828). In the main analysis, the median follow-up duration was 5.6 years in the EoE cohort and 6.9 years in the control cohort.

### 3.3. Sensitivity Analysis

In the sensitivity analysis applying different EoE definitions, increased risks of coded ocular diagnostic categories were observed among individuals with EoE (Table S3). Visual disturbance and blindness showed significant HRs of 1.783 (Definition 1) and 1.963 (Definition 2), followed by disorders of refraction and accommodation (HRs: 1.605 and 1.740), disorders of the lacrimal system (HRs: 1.909 and 1.955), blepharitis (HRs: 1.651 and 1.983), ocular pain (HRs: 1.418 and 1.623), cataract (HRs: 1.888 and 2.026), glaucoma (HRs: 1.862 and 1.993), and disorders of the vitreous body and globe (HRs: 2.293 and 2.550).

Similarly, in the sensitivity analysis applying 5- and 10-year follow-up periods (Table S4), the risks of developing visual disturbance and blindness (HRs: 1.359 and 1.539, respectively), disorders of refraction and accommodation (HRs: 1.176 and 1.265), cataract (HRs: 1.488 and 1.491), and disorders of the vitreous body and globe (HRs: 1.475 and 1.796) in the EoE group were also significant. Additionally, after applying washout periods of 12, 24, and 36 months (Table S5), the risks of visual disturbance and blindness, disorders of refraction and accommodation, disorders of the lacrimal system, cataract, glaucoma, and disorders of the vitreous body and globe remained significant. In analyses matching for age and sex, visual disturbance and blindness, disorders of refraction and accommodation, disorders of the lacrimal system, cataract, and disorders of the vitreous body and globe also showed elevated risks in patients with EoE. Similar trends were observed when the matching covariates included age at index, sex, socioeconomic status, mental and behavioral disorders due to psychoactive substance use, and medical utilization status (Table S6). To partially address surveillance bias, we conducted an additional sensitivity analysis that incorporated a history of encounters for examination of the eyes and vision (ICD-10-CM: Z01.0) as an additional covariate (Table S6, Model 3). The results showed that patients with EoE remained at an elevated risk of glaucoma and cataract in this model. However, associations with disorders of refraction and accommodation, hordeolum of the eyelid, blepharitis, ocular pain, and disorders of the sclera were attenuated after accounting for eye examination history.

Because residual imbalance remained for asthma, allergic rhinitis, and non-infective enteritis and colitis after matching, we performed additional exclusion-based sensitivity analyses to evaluate the potential influence of these comorbidities. These analyses were intended to provide a supplementary assessment of residual confounding rather than to fully replace covariate adjustment or matching. After excluding patients with asthma from both the EoE and control groups, the risks of developing visual disturbance and blindness, disorders of the lacrimal system, cataract, and disorders of the vitreous body and globe remained significantly increased in patients with EoE. A similar trend was observed after excluding patients with non-infective enteritis and colitis. However, after excluding patients with allergic rhinitis, only visual disturbance and blindness and disorders of the vitreous body and globe remained significant. In this model, the associations between EoE and disorders of refraction and accommodation, disorders of the lacrimal system, hordeolum of the eyelid, blepharitis, ocular pain, and cataract were no longer significant (Table S7).

### 3.4. Stratification Analysis

The significantly increased risk of several coded ocular diagnostic categories in patients with EoE remained evident in the stratified analyses by sex, age, and race. When stratified by sex (Table 2 and Table 3), visual disturbance and blindness (HR: 1.360 and 1.809, respectively), disorders of refraction and accommodation (HR: 1.337 and 1.243), disorders of the lacrimal system (HR: 1.541 and 1.690), cataract (HR: 1.431 and 1.439), and disorders of the vitreous body and globe (HR: 1.561 and 2.252) showed increased risks in both male and female patients with EoE compared with their corresponding control groups, while glaucoma (HR: 1.531) also showed a significantly elevated risk in females. This trend remained consistent in the age-stratified analysis, with an increased risk of developing coded ocular diagnostic categories among patients with EoE aged 18–64 years and those aged >65 years (Table 4 and Table 5). In the stratified analysis by race, White patients with EoE exhibited a notably higher risk of developing visual disturbance and blindness (HR: 1.485), disorders of refraction and accommodation (HR: 1.410), disorders of the lacrimal system (HR: 1.806), ocular pain (HR: 1.431), cataract (HR: 1.429), glaucoma (HR: 1.414), and disorders of the vitreous body and globe (HR: 1.848) compared with the White control cohort. Nevertheless, only visual disturbance and blindness showed a significant HR of 1.481 in Black patients with EoE (Table 6 and Table 7).

## 4. Discussion

In this large-scale EHR-based cohort study, EoE diagnostic coding was associated with a higher subsequent occurrence of several coded ocular diagnostic categories, including visual disturbance and blindness, disorders of refraction and accommodation, disorders of the lacrimal system, cataract, glaucoma, and disorders of the vitreous body and globe. These findings should be interpreted as associations between coded EoE diagnoses and subsequent coded ocular diagnoses rather than as evidence that EoE directly causes ocular disease. The clinical specificity of the outcomes also varied. Cataract, glaucoma, and disorders of the vitreous body and globe may have clearer long-term ophthalmologic relevance, whereas broader categories such as visual disturbance, disorders of refraction and accommodation, and ocular pain may be more susceptible to differences in detection patterns, coding practices, and healthcare utilization.

EoE, as a chronic inflammatory disease, shares related pathogenic mechanisms with psoriasis and HS. Previous research has shown that fibrosis and stricture are common manifestations in patients with EoE, while fibroblasts secrete tumor necrosis factor (TNF)-α to induce epithelial lysyl oxidase, a collagen cross-linking enzyme [14]. Other studies have also revealed that TNF-α levels are significantly increased in patients with psoriasis and HS [15,16]. Moreover, in patients with HS, the risk of developing ocular disorders such as glaucoma, lacrimal system disorders, ocular pain, visual disturbances, and disorders of refraction and accommodation was significantly increased [11]. In patients with psoriasis, a higher risk of retinal detachment and retinal vascular occlusion has been demonstrated, and psoriasis has also been associated with ocular diseases such as conjunctivitis, episcleritis, and uveitis [17,18]. Based on the relationships among EoE, HS, psoriasis, and ocular diseases, our findings regarding the association between EoE and coded ocular diagnostic categories may be biologically plausible, although the exact underlying mechanisms remain unclear.

Several biological pathways may provide possible hypotheses for selected associations; however, these mechanisms remain speculative in the present EHR-based observational design. In view of our findings, several hypotheses are suggested. First, an elevated risk of cataract has been observed in patients with psoriasis, which shares similar inflammatory mechanisms with EoE. The inflammatory pathogenesis involves mediators including C-reactive protein (CRP), intracellular adhesion molecule-1 (ICAM-1), interleukin (IL)-1β, IL-6, IL-12, and IL-23, which have been demonstrated to be associated with an increased risk of cataract. This relationship might explain the potential association between EoE and cataract formation [19]. Similarly, the stability of the vitreous body is related to collagen, hyaluronic acid, and the extracellular matrix. The inflammatory response in EoE may induce tissue remodeling, fibroblast activation, and extracellular matrix changes, which could explain the higher risk of disorders of the vitreous body and refraction in patients with EoE [1]. Additionally, oxidative stress has been reported to be triggered in EoE [20]. Oxidative stress could induce mitochondrial DNA damage and retinal ganglion cell (RGC) loss, potentially leading to glaucoma and visual disturbance [21]. Regarding TNF-α, which is secreted by epithelial cells and fibroblasts, it plays an important role in EoE pathogenesis [22]. A study by Qian Li et al. demonstrated that transient receptor potential vanilloid 4 (TRPV4), which is distributed in the retina, might elevate TNF-α levels and thereby exacerbate RGC apoptosis [23]. Under inflammatory conditions, endothelial dysfunction may occur, thereby damaging the vascular system and decreasing retinal perfusion, which could contribute to various ocular diseases [24]. Moreover, dry eye disease (DED), one of the disorders of the lacrimal system, is characterized by inflammation, mitochondrial dysfunction, and oxidative stress, which are also observed in EoE [25]. In another study, blepharitis, a chronic inflammatory condition, was associated with meibomian gland dysfunction (MGD), which accounts for more than 70% of DED cases [26]. These findings suggest potentially shared mechanisms between coded ocular diagnostic categories and EoE. Furthermore, regarding microbiota patterns, EoE has been found to exhibit a distinct esophageal microbiota compared with healthy individuals, including Proteobacteria such as Neisseria and Corynebacterium [27]. Among ocular microorganisms, numerous bacterial species overlapping with esophageal microorganisms have also been identified [28]. These microorganisms could trigger intraocular inflammation, which might be associated with EoE, although the exact mechanisms still require further investigation.

The additional sensitivity analyses provide important context for interpreting these findings. The model incorporating prior eye and vision examination was intended to partially address surveillance bias related to ophthalmologic evaluation. In this model, elevated associations remained mainly for glaucoma and cataract, suggesting that these outcomes may be less dependent on differential opportunities for eye-related diagnostic coding than some broader categories. In contrast, after excluding allergic rhinitis, only visual disturbance and blindness and disorders of the vitreous body and globe remained significant. This attenuation suggests that atopic comorbidity, allergy-related healthcare utilization, or related treatment patterns may partly explain some of the observed associations. Therefore, the current results should not be interpreted as uniformly robust across all ocular outcomes. These residual imbalances, particularly for atopic and inflammatory comorbidities, further support interpreting the findings as exploratory associations rather than causal effects.

The present study should not be interpreted as demonstrating that EoE directly manifests in the eye. EoE and ocular diagnostic categories were treated as distinct clinical entities in the study design, with EoE diagnostic coding defining the exposure and subsequent coded ocular diagnoses defining the outcomes. Therefore, the findings are better understood as an exploratory assessment of ocular diagnostic burden after EoE coding rather than evidence of extra-esophageal ophthalmic manifestations of EoE.

Our study has several strengths. First, we used propensity score matching to reduce potential confounding and to provide a more precise assessment of the relationship between EoE and coded ocular diagnostic categories. Second, sensitivity analyses using different definitions of EoE, follow-up durations, washout periods, and matching covariates substantially enhanced the credibility of our findings. Moreover, stratified analyses supported the consistency of the observed associations across different subgroups. However, several limitations should be acknowledged. First, due to the retrospective design of this study, a direct causal relationship between EoE and coded ocular diagnostic categories could not be established. Second, though adopting the Global Collaborative Network, the TriNetX database is derived from mostly US-based healthcare institutions, which may limit the generalizability of the findings to the broader population. Third, detection and surveillance bias may have influenced the results. Patients with EoE are more likely to engage in healthcare visits, which could increase the likelihood of detecting ocular disorders. In contrast, individuals in the control group may have lower healthcare utilization, resulting in fewer opportunities for ophthalmologic diagnoses. These potential sources of surveillance bias may have affected the observed incidence of coded ocular diagnostic categories and should be considered when interpreting the findings. Fourth, formal interaction testing between subgroup variables was not available within the TriNetX analytics platform. Therefore, subgroup findings should be interpreted as exploratory rather than confirmatory. In particular, several race-stratified analyses involved relatively low event counts, which may have limited statistical power and contributed to wider confidence intervals in certain subgroups. Fifth, because administrative data were used, misclassification and underreporting of both EoE and coded ocular diagnostic categories could not be completely eliminated. Such misclassification bias, inherent to a database-driven design, may have influenced outcome assessment. In addition, due to the lack of specialist-level clinical information within the TriNetX database, it was not possible to verify whether all diagnoses were made by relevant specialists, which may have affected outcome accuracy. Sixth, residual confounding remains an important limitation. Although the primary propensity score model included demographic variables, body mass index, selected cardiometabolic comorbidities, proton pump inhibitor use, systemic corticosteroid use, socioeconomic vulnerability, substance-related disorders, and healthcare utilization variables, post-matching imbalance persisted for asthma, allergic rhinitis, and non-infective enteritis and colitis. These conditions are clinically relevant because they may reflect atopy, systemic inflammation, corticosteroid exposure, healthcare-seeking behavior, and the likelihood of EoE ascertainment or ocular diagnostic coding. Due to a system-defined covariate limit within the TriNetX analytics platform, we were unable to include all of these variables simultaneously in the primary propensity score model. We therefore performed exclusion-based sensitivity analyses to evaluate the potential influence of these residual imbalances. However, such analyses cannot fully substitute for comprehensive covariate adjustment or matching. Accordingly, the observed associations should be interpreted cautiously and should not be considered evidence of causal effects. In addition, some potentially important confounders were unavailable. Both EoE and ocular disorders have been associated with food and environmental allergens as well as genetic factors, whereas environmental exposures and genetic predisposition could not be accounted for in the present study [29,30]. Seventh, regarding outcome definitions, several ocular outcome categories included clinically heterogeneous conditions and may have encompassed diagnoses with varying clinical severity and specificity. Some outcomes may also have been more susceptible to incidental detection during routine clinical encounters. Nevertheless, these broad ICD-10-CM-based categories were intentionally adopted to characterize the overall ocular comorbidity profile associated with EoE in a large-scale real-world database setting. Eighth, statistical reporting was also limited by the TriNetX analytics environment. The platform does not provide detailed person-time calculations, incidence rates per person-year, or formal proportional hazards diagnostics such as Schoenfeld residual analyses. In addition, some implementation details of matching and survival modeling are platform-defined. Therefore, the HRs reported in this study should be interpreted as relative measures of association rather than estimates of absolute clinical risk. The absence of incidence rates and absolute risk differences limits assessment of the clinical magnitude of the observed associations. Ninth, multiple testing should also be considered when interpreting the findings. The current study examined several ocular diagnostic categories across multiple sensitivity and subgroup analyses. Because no formal correction for multiple comparisons was applied, the reported nominal *p*-values and confidence intervals should be interpreted cautiously. The findings of the current study should be interpreted in the context of these limitations inherent to retrospective EHR-based observational research and should be viewed as exploratory and hypothesis-generating rather than confirmatory.

The possibility that a combination of biases contributed to the observed associations cannot be excluded. Surveillance bias, residual confounding by atopic or inflammatory comorbidities, medication exposure, and diagnostic coding practices may have influenced the results, particularly for broad and detection-prone outcomes. However, these limitations should be viewed as factors that define the interpretive boundary of the study rather than as reasons to dismiss the findings. We performed several analyses to evaluate major sources of bias, including exclusion of prior ocular diagnoses, washout periods, adjustment for eye and vision examination history, alternative EoE definitions, and exclusion-based analyses for selected residual comorbidities. These approaches cannot eliminate all unmeasured bias, but they provide a structured assessment of robustness. Accordingly, the findings should be interpreted as exploratory real-world evidence requiring external validation rather than as definitive causal evidence.

From a scientific perspective, the novelty of this study lies in its characterization of the longitudinal ocular diagnostic profile of patients with EoE in a large-scale real-world cohort. Previous evidence has mainly focused on allergic comorbidities of EoE or isolated ocular conditions, whereas population-level data on subsequent coded ocular diagnoses after EoE diagnosis remain limited. By evaluating multiple ocular diagnostic categories and applying several sensitivity analyses, the present study provides an initial signal that EoE diagnostic coding may identify a patient population with a higher subsequent ocular diagnostic burden. From a clinical perspective, the findings should be interpreted as a signal for clinical awareness rather than as an indication for universal ophthalmologic screening. Clinicians caring for patients with EoE should not infer that EoE commonly manifests in the eye. However, when patients with EoE report persistent visual symptoms, ocular discomfort, or recurrent eye-related complaints, clinicians may consider timely ophthalmologic evaluation according to usual clinical indications. This may be particularly relevant for patients with substantial atopic comorbidity, frequent healthcare utilization, corticosteroid exposure, older age, or other established ophthalmologic risk factors. Thus, the practical value of the study is not to establish a new screening recommendation, but to highlight a potential ocular diagnostic burden that may need attention in selected clinical contexts.

## 5. Conclusions

In conclusion, this large-scale EHR-based cohort study found that EoE diagnostic coding was associated with a higher subsequent occurrence of several coded ocular diagnostic categories. However, the robustness of these associations differed across outcomes and sensitivity analyses. Cataract, glaucoma, and disorders of the vitreous body and globe appeared more clinically relevant in selected models, whereas broader or detection-prone categories should be interpreted with greater caution. Because potential surveillance bias, residual confounders related to atopic comorbidity, outcome misclassification, and limited availability of absolute risk estimates remain important limitations, these findings should be considered exploratory and hypothesis-generating. Further studies with validated EoE definitions, ophthalmologist-confirmed ocular outcomes, and detailed healthcare utilization measures are needed to clarify the clinical relevance of these associations.

## Figures and Tables

**Table 1:** Baseline characteristics.

Covariates	Before Matching			After Matching		
	EoE Cohort (*n* = 66,282)	Control Cohort (*n* = 8,395,223)	SMD	EoE Cohort (*n* = 64,613)	Control Cohort (*n* = 64,613)	SMD
**Age at index**						
Mean ± SD	29.9 ± 16.5	39.1 ± 17.9	0.54	29.9 ± 16.5	29.9 ± 16.5	0.00
**Sex**						
Male	40,579 (62.6)	3,748,465 (45.9)	0.34	40,392 (62.5)	40,261 (62.3)	0.00
Female	24,189 (37.3)	4,387,855 (53.8)	0.33	24,189 (37.4)	24,178 (37.4)	0.00
Unknown Gender	32 (<0.1)	24,646 (0.3)	0.06	32 (0.1)	174 (0.3)	0.06
**Race, n (%)**						
White	52,909 (81.7)	4,531,981 (55.5)	0.59	52,722 (81.6)	52,750 (81.6)	0.00
Black or African American	3063 (4.7)	855,539 (10.5)	0.22	3063 (4.7)	3041 (4.7)	0.00
Asian	909 (1.4)	341,252 (4.2)	0.17	909 (1.4)	1155 (1.8)	0.03
American Indian or Alaska Native	192 (0.3)	23,486 (0.3)	0.00	192 (0.3)	95 (0.1)	0.03
Native Hawaiian or Other Pacific Islander	105 (0.2)	19,441 (0.2)	0.02	105 (0.2)	80 (0.1)	0.01
Other Race	2124 (3.3)	434,590 (5.3)	0.10	2124 (3.3)	1475 (2.3)	0.06
Unknown Race	5498 (8.5)	1,954,677 (24.0)	0.43	5498 (8.5)	6017 (9.3)	0.03
**Socioeconomic status**						
Persons with potential health hazards related to socioeconomic and psychosocial circumstances	121 (0.2)	20,460 (0.3)	0.01	121 (0.2)	121 (0.2)	0.00
**Lifestyle**						
Mental and behavioral disorders due to psychoactive substance use	1045 (1.6)	154,324 (1.9)	0.02	1045 (1.6)	1028 (1.6)	0.00
**Medical Utilization**						
Visit: Ambulatory	35,583 (54.9)	5,019,474 (61.5)	0.13	35,451 (54.9)	36,730 (56.8)	0.04
Visit: Inpatient Encounter	2837 (4.4)	327,773 (4.0)	0.02	2824 (4.4)	3504 (5.4)	0.05
**BMI**						
Greater than 25 kg/m^2^	9061 (14.0)	1,107,047 (13.6)	0.01	9057 (14.0)	9037 (14.0)	0.00
**Comorbidities**						
Asthma	3575 (5.5)	119,712 (1.5)	0.22	3558 (5.5)	1247 (1.9)	**0.19**
Atopic dermatitis	300 (0.5)	10,925 (0.1)	0.06	300 (0.5)	108 (0.2)	0.05
Vasomotor and allergic rhinitis	2755 (4.3)	127,582 (1.6)	0.16	2741 (4.2)	1224 (1.9)	**0.14**
Essential hypertension	1879 (2.9)	406,654 (5.0)	0.11	1879 (2.9)	1884 (2.9)	0.00
Hyperlipidemia,	952 (1.5)	174,960 (2.1)	0.05	952 (1.5)	965 (1.5)	0.00
Diabetes mellitus	638 (1.0)	143,128 (1.8)	0.07	638 (1.0)	648 (1.0)	0.00
Cerebrovascular diseases	173 (0.3)	32,906 (0.4)	0.02	173 (0.3)	205 (0.3)	0.01
Chronic kidney disease	106 (0.2)	21,684 (0.3)	0.02	105 (0.2)	110 (0.2)	0.00
Noninfective enteritis and colitis	1689 (2.6)	44,763 (0.5)	0.17	1686 (2.6)	544 (0.8)	**0.14**
**Comedications**						
Proton pump inhibitors	8118 (12.5)	175,147 (2.1)	0.41	7931 (12.3)	7928 (12.3)	0.00
Systemic corticosteroids	4110 (6.3)	273,008 (3.3)	0.14	4081 (6.3)	2954 (4.6)	0.08
Dupilumab	125 (0.2)	284 (<0.1)	0.06	125 (0.2)	10 (<0.1)	0.06

Legends: EoE, eosinophilic esophagitis; 95% CI, 95% confidence interval; HR, hazard ratio. In the TriNetX analytics platform, to protect patient privacy, any incident case count of 10 or fewer is reported as 10. Propensity score matching was reperformed for each model, with matched covariates including age, sex, race, body mass index, comorbidity status (diabetes mellitus, hypertension, and hyperlipidemia), comedication use (proton pump inhibitors), mental and behavioral disorders due to psychoactive substance use, and socioeconomic factors (persons with potential health hazards related to socioeconomic and psychosocial circumstances). The follow-up period was set at up to 15 years after the index date, and the washout period was set at 3 months.

**Table 2:** Sex-based stratification analysis in ocular comorbidities (male).

Outcomes	Male		
	EoE Cohort (%)	Control Cohort (%)	HR (95% CI)
Visual disturbance and blindness	474 (1.2)	411 (1.0)	1.36 (1.191, 1.552)
Disorders of refraction and accommodation	430 (1.1)	372 (0.9)	1.337 (1.163, 1.536)
Disorders of lacrimal system	143 (0.4)	107 (0.3)	1.541 (1.199, 1.981)
Hordeolum of eyelid	92 (0.2)	126 (0.3)	0.866 (0.661, 1.133)
Blepharitis	51 (0.1)	50 (0.1)	1.181 (0.799, 1.746)
Ocular pain	91 (0.2)	87 (0.2)	1.226 (0.914, 1.646)
Cataract	187 (0.5)	156 (0.4)	1.431 (1.157, 1.771)
Disorders of sclera	NA		
Glaucoma	92 (0.2)	83 (0.2)	1.312 (0.975, 1.767)
Disorders of vitreous body and globe	94 (0.2)	71 (0.2)	1.561 (1.146, 2.125)

EoE, eosinophilic esophagitis; 95% CI, 95% confidence interval; HR, hazard ratio.

**Table 3:** Sex-based stratification analysis in ocular comorbidities (female).

Outcomes	Female		
	EoE Cohort (%)	Control Cohort (%)	HR (95% CI)
Visual disturbance and blindness	495 (2.0)	326 (1.3)	1.809 (1.573, 2.081)
Disorders of refraction and accommodation	282 (1.2)	261 (1.1)	1.243 (1.05, 1.472)
Disorders of lacrimal system	177 (0.7)	124 (0.5)	1.69 (1.343, 2.127)
Hordeolum of eyelid	83 (0.3)	75 (0.3)	1.322 (0.967, 1.807)
Blepharitis	47 (0.2)	40 (0.2)	1.373 (0.9, 2.095)
Ocular pain	71 (0.3)	68 (0.3)	1.233 (0.884, 1.721)
Cataract	139 (0.6)	116 (0.5)	1.439 (1.124, 1.842)
Disorders of sclera	NA		
Glaucoma	65 (0.3)	50 (0.2)	1.531 (1.058, 2.215)
Disorders of vitreous body and globe	93 (0.4)	49 (0.2)	2.252 (1.593, 3.185)

EoE, eosinophilic esophagitis; 95% CI, 95% confidence interval; HR, hazard ratio.

**Table 4:** Age-based stratification analysis in ocular comorbidities (18 to 64 years old).

Outcomes	18 to 64 Years Old		
	EoE Cohort (%)	Control Cohort (%)	HR (95% CI)
Visual disturbance and blindness	834 (1.4)	710 (1.2)	1.396 (1.263, 1.543)
Disorders of refraction and accommodation	609 (1.0)	599 (1.0)	1.177 (1.051, 1.318)
Disorders of lacrimal system	247 (0.4)	210 (0.4)	1.378 (1.146, 1.657)
Hordeolum of eyelid	163 (0.3)	202 (0.3)	0.967 (0.786, 1.189)
Blepharitis	77 (0.1)	78 (0.1)	1.161 (0.847, 1.591)
Ocular pain	148 (0.2)	164 (0.3)	1.077 (0.862, 1.346)
Cataract	141 (0.2)	101 (0.2)	1.675 (1.296, 2.163)
Disorders of sclera	18 (<0.1)	14 (<0.1)	1.495 (0.743, 3.01)
Glaucoma	100 (0.2)	109 (0.2)	1.082 (0.824, 1.42)
Disorders of vitreous body and globe	125 (0.2)	97 (0.2)	1.532 (1.174, 1.998)

EoE, eosinophilic esophagitis; 95% CI, 95% confidence interval; HR, hazard ratio.

**Table 5:** Age-based stratification analysis in ocular comorbidities (greater than 65 years old).

Outcomes	Greater Than 65 Years Old		
	EoE Cohort (%)	Control Cohort (%)	HR (95% CI)
Visual disturbance and blindness	136 (2.8)	83 (1.7)	1.75 (1.332, 2.299)
Disorders of refraction and accommodation	102 (2.1)	54 (1.1)	2.007 (1.443, 2.791)
Disorders of lacrimal system	72 (1.5)	42 (0.9)	1.826 (1.248, 2.671)
Hordeolum of eyelid	12 (0.2)	18 (0.4)	0.712 (0.343, 1.477)
Blepharitis	21 (0.4)	11 (0.2)	2.04 (0.983, 4.23)
Ocular pain	14 (0.3)	13 (0.3)	1.144 (0.538, 2.434)
Cataract	185 (3.8)	137 (2.8)	1.442 (1.156, 1.798)
Disorders of sclera	NA		
Glaucoma	57 (1.2)	40 (0.8)	1.51 (1.008, 2.263)
Disorders of vitreous body and globe	62 (1.3)	38 (0.8)	1.738 (1.161, 2.603)

EoE, eosinophilic esophagitis; 95% CI, 95% confidence interval; HR, hazard ratio.

**Table 6:** Race-based stratification analysis in ocular comorbidities.

Outcomes	White		
	EoE Cohort (%)	Control Cohort (%)	HR (95% CI)
Visual disturbance and blindness	820 (1.6)	676 (1.3)	1.485 (1.341, 1.644)
Disorders of refraction and accommodation	579 (1.1)	489 (0.9)	1.41 (1.25, 1.591)
Disorders of lacrimal system	260 (0.5)	175 (0.3)	1.806 (1.49, 2.188)
Hordeolum of eyelid	145 (0.3)	170 (0.3)	1.065 (0.853, 1.33)
Blepharitis	74 (0.1)	84 (0.2)	1.085 (0.794, 1.484)
Ocular pain	136 (0.3)	116 (0.2)	1.431 (1.117, 1.834)
Cataract	257 (0.5)	222 (0.4)	1.429 (1.194, 1.711)
Disorders of sclera	NA		
Glaucoma	109 (0.2)	94 (0.2)	1.414 (1.073, 1.865)
Disorders of vitreous body and globe	148 (0.3)	98 (0.2)	1.848 (1.431, 2.387)

EoE, eosinophilic esophagitis; 95% CI, 95% confidence interval; HR, hazard ratio.

**Table 7:** Race-based stratification analysis in ocular comorbidities^a^.

Outcomes	Black		
	EoE Cohort (%)	Control Cohort (%)	HR (95% CI)
Visual disturbance and blindness	64 (2.1)	51 (1.7)	1.481 (1.024, 2.141)
Disorders of refraction and accommodation	43 (1.4)	47 (1.5)	1.08 (0.714, 1.634)
Disorders of lacrimal system	19 (0.6)	17 (0.6)	1.337 (0.694, 2.576)
Hordeolum of eyelid	NA		
Blepharitis	NA		
Ocular pain	13 (0.4)	16 (0.5)	0.978 (0.469, 2.036)
Cataract	28 (0.9)	21 (0.7)	1.606 (0.911, 2.831)
Disorders of sclera	NA		
Glaucoma	24 (0.8)	16 (0.5)	1.812 (0.962, 3.416)
Disorders of vitreous body and globe	NA		

EoE, eosinophilic esophagitis; 95% CI, 95% confidence interval; HR, hazard ratio.

## Data Availability

Data in this study were retrieved from TriNetX Research Network. All data available in the database were administrated by the TriNetX platform. Detailed information can be retrieved at the official website of the research network (https://trinetx.com; Accessed date: 1 July 2026).

## Supplementary Materials

The following are available online at https://www.mdpi.com/article/10.3390/life16071156/s1, Table S1: Utilized proxy codes, Table S2: Description of applied sensitivity analysis models, Table S3: Sensitivity analysis of applying different EoE definitions, Table S4: Sensitivity analysis of applying different follow-up period, Table S5: Sensitivity analysis of applying different wash-out period after index date, Table S6: Sensitivity analysis of applying different matching covariates, Table S7: Sensitivity analysis of excluding for critical confounders.
