# Supplementary material for: Association Between Eosinophilic Esophagitis and Coded Ocular Diagnoses: A Retrospective Cohort Study
Source: Life (Basel). 2026 Jul 13;16(7):1156. doi: 10.3390/life16071156 (PMC13412847; doi:10.3390/life16071156)
Supplement: Supplementary file 1 [file life-16-01156-s001.zip › life-4294539-supplementary.pdf]

Supplementary Files

Table S1. Utilized proxy codes [a]

**Description**	**ICD-10-CM codes**
Study population	
Eosinophilic esophagitis	ICD-10-CM K20.0
Outcome events	
Visual disturbance and blindness	ICD-10-CM: H53-H54
Disorders of refraction and accommodation	ICD-10-CM: H52
Disorders of lacrimal system	ICD-10-CM: H04
Hordeolum of eyelid	ICD-10-CM: H00.0
Blepharitis	ICD-10-CM: H01.0
Ocular pain	ICD-10-CM: H57.1
Cataract	ICD-10-CM: H25,H26,H28
Disorders of sclera	ICD-10-CM: H15
Glaucoma	ICD-10-CM: H40-H42
Disorders of vitreous body and globe	ICD-10-CM: H43-H44
Covariates and other definitions	
Essential hypertension	ICD-10-CM: I10
Hyperlipidemia	ICD-10-CM: E78.5
Diabetes mellitus	ICD-10-CM: E08-E15
Asthma	ICD-10-CM: J45

Atopic dermatitis	ICD-10-CM: L20
Vasomotor and allergic rhinitis	ICD-10-CM: J30
Cerebrovascular diseases	ICD-10-CM: I60-I69
Chronic kidney disease	ICD-10-CM: N18
Noninfective enteritis and colitis	ICD-10-CM: K50-K52
Persons with potential health hazards related to socioeconomic and psychosocial circumstances	ICD-10-CM: Z55-Z65
Mental and behavioral disorders due to psychoactive substance use	ICD-10-CM: F10-F19
Encounter for general adult examination	ICD-10-CM: Z00.0
Encounter for examination of eyes and vision	ICD-10-CM: Z01.0

[a]ICD-10-CM: International Classification of Diseases, Tenth Revision, Clinical Modification

Table S2. Description of applied sensitivity analysis models

Models	Description
**Legends**	EoE, eosinophilic esophagitis; 95% CI, 95% confidence interval; HR, hazard ratio. In the TriNetX analytics platform, to protect patient privacy, any incident case count of 10 or fewer is reported as 10.Propensity score matching was reperformed in each models, with the matched covariates including age, sex, race, body mass index, status of comorbidities (diabetes mellitus, hypertension, hyperlipidemia), status of comedication use (proton pump inhibitors), mental and behavioral disorders due to psychoactive substance use, socioeconomic factors (persons with potential health hazards related to socioeconomic and psychosocial circumstances). Follow-up period was set as up to 15 years after index date, and wash-out period was set as 3 months.
**Applying multiple proxy-based criteria to define eosinophilic esophagitis (EoE).**	
Algorithm 1	Patients were classified into the EoE group only if they had a documented diagnosis of ICD-10-CM code K20.0 (eosinophilic esophagitis) and a corresponding prescription for proton pump inhibitors (ATC code A02BC).
Algorithm 2	Patients were classified into the EoE group only if they had a documented diagnosis of ICD-10-CM code K20.0 (eosinophilic esophagitis) and esophagogastroduodenoscopy procedures (CPT code: 1021431)
**Applying varying washout periods following the index date.**	
12 months/24 months after index date	Events that took place within the predefined washout periods were excluded from further analysis. Additionally, all subgroup analyses were conducted using a uniform follow-up period of up to 15 years.

5 years /10 years /15 years after index date	The analysis considered only those outcome events that arose during the specified follow-up window. Furthermore, a three-month washout periods was consistently implemented across all subgroup analyses.
**Applying alternative sets of covariates for matching.**	
Matching model 1	Matching covariates include age at index and sex
Matching model 2	Matching covariates include age at index, sex, socioeconomic status, mental and behavioral disorders due to psychoactive substance use and medical utilization status
Matching model 3	Matching covariates included those used in the main analysis: age, sex, race, body mass index, comorbidity status (diabetes mellitus, hypertension, and hyperlipidemia), concomitant medication use (proton pump inhibitors), mental and behavioral disorders due to psychoactive substance use, socioeconomic factors (individuals with potential health hazards related to socioeconomic and psychosocial circumstances), and history of encounters for examination of the eyes and vision (ICD-10-CM: Z01.0).
**Excluding for critical confounders**	
Exclude for asthma/allergic rhinitis/non-infective enteritis/colitis	To avoid potential confounding bias caused by asthma, allergic rhinitis and non-infective enteritis/colitis, which is common in EoE, patients with any records of being diagnosed of asthma, allergic rhinitis and non-infective enteritis/colitis will be excluded from EoE group and control group.

Table S3. Sensitivity analysis of applying different EoE definitions

Outcomes	Definition 1			Definition 2		
	EoE cohort (%)[a]	Control cohort (%)	HR (95% CI)[a]	EoE cohort (%)[a]	Control cohort (%)	HR (95% CI)[a]
Visual disturbance and blindness	932 (2.0)	566 (1.2)	1.783 (1.606,1.979)	753 (2.1)	433 (1.2)	1.963 (1.744,2.21)
Disorders of refraction and accommodation	676 (1.5)	446 (1.0)	1.605 (1.424,1.809)	548 (1.5)	347 (1.0)	1.74 (1.521,1.991)
Disorders of lacrimal system	328 (0.7)	184 (0.4)	1.909 (1.593,2.287)	231 (0.6)	132 (0.4)	1.955 (1.578,2.422)
Hordeolum of eyelid	165 (0.4)	156 (0.3)	1.142 (0.917,1.422)	140 (0.4)	125 (0.3)	1.269 (0.997,1.616)
Blepharitis	91 (0.2)	59 (0.1)	1.651 (1.189,2.291)	77 (0.2)	43 (0.1)	1.983 (1.365,2.881)
Ocular pain	166 (0.4)	127 (0.3)	1.418 (1.125,1.787)	135 (0.4)	94 (0.3)	1.623 (1.247,2.113)
Cataract	287 (0.6)	165 (0.4)	1.888 (1.558,2.286)	210 (0.6)	117 (0.3)	2.026 (1.616,2.541)
Disorders of sclera	22 (<0.1)	12 (<0.1)	1.949 (0.964,3.941)		NA	
Glaucoma	155 (0.3)	89 (0.2)	1.862 (1.434,2.417)	119 (0.3)	67 (0.2)	1.993 (1.477,2.69)
Disorders of vitreous body and globe	190 (0.4)	89 (0.2)	2.293 (1.782,2.95)	138 (0.4)	61 (0.2)	2.55 (1.886,3.448)

EoE, eosinophilic esophagitis; 95% CI, 95% confidence interval; HR, hazard ratio. In the TriNetX analytics platform, to protect patient privacy, any incident case count of 10 or fewer is reported as 10.Propensity score matching was reperformed in each models, with the matched covariates including age, sex, race, body mass index, status of comorbidities (diabetes mellitus, hypertension, hyperlipidemia), status of comedication use (proton pump inhibitors), mental and behavioral disorders due to psychoactive substance use, socioeconomic factors (persons with potential health hazards related to socioeconomic and psychosocial circumstances)

Table S4. Sensitivity analysis of applying different follow-up period

Outcomes	Up to 5 years			Up to 10 years		
	EoE cohort (%)[a]	Control cohort (%)	HR (95% CI)[a]	EoE cohort (%)[a]	Control cohort (%)	HR (95% CI)[a]
Visual disturbance and blindness	416 (0.6)	327 (0.5)	1.359 (1.176,1.571)	781 (1.2)	575 (0.9)	1.539 (1.381,1.714)
Disorders of refraction and accommodation	363 (0.6)	328 (0.5)	1.176 (1.013,1.366)	603 (0.9)	533 (0.8)	1.265 (1.126,1.421)
Disorders of lacrimal system	152 (0.2)	138 (0.2)	1.176 (0.934,1.481)	252 (0.4)	207 (0.3)	1.364 (1.135,1.64)
Hordeolum of eyelid	59 (0.1)	86 (0.1)	0.733 (0.527,1.021)	126 (0.2)	152 (0.2)	0.938 (0.741,1.189)
Blepharitis	39 (0.1)	37 (0.1)	1.129 (0.72,1.77)	79 (0.1)	71 (0.1)	1.27 (0.921,1.75)
Ocular pain	63 (0.1)	71 (0.1)	0.947 (0.675,1.33)	131 (0.2)	123 (0.2)	1.206 (0.943,1.542)
Cataract	130 (0.2)	93 (0.1)	1.488 (1.14,1.942)	251 (0.4)	191 (0.3)	1.491 (1.235,1.801)
Disorders of sclera		NA		17 (<0.1)	16 (<0.1)	1.191 (0.601,2.359)
Glaucoma	68 (0.1)	61 (0.1)	1.188 (0.841,1.678)	115 (0.2)	109 (0.2)	1.187 (0.913,1.543)
Disorders of vitreous body and globe	76 (0.1)	55 (0.1)	1.475 (1.043,2.087)	145 (0.2)	91 (0.1)	1.796 (1.381,2.334)

EoE, eosinophilic esophagitis; 95% CI, 95% confidence interval; HR, hazard ratio. In the TriNetX analytics platform, to protect patient privacy, any incident case count of 10 or fewer is reported as 10.Propensity score matching was reperformed in each models, with the matched covariates including age, sex, race, body mass index, status of comorbidities (diabetes mellitus, hypertension, hyperlipidemia), status of comedication use (proton pump inhibitors), mental and behavioral disorders due to psychoactive substance use, socioeconomic factors (persons with potential health hazards related to socioeconomic and psychosocial circumstances)

Table S5. Sensitivity analysis of applying different wash-out period after index date

Outcomes	12 months of wash-out period			24 months of wash-out period			36 months of wash-out period		
	EoE cohort (%)[a]	Control cohort (%)	HR (95% CI)[a]	EoE cohort (%)[a]	Control cohort (%)	HR (95% CI)[a]	EoE cohort (%)[a]	Control cohort (%)	HR (95% CI)[a]
Visual disturbance and blindness	911 (1.4)	702 (1.1)	1.567 (1.42,1.73)	836 (1.3)	630 (1.0)	1.628 (1.468,1.806)	767 (1.2)	570 (0.9)	1.675 (1.502,1.867)
Disorders of refraction and accommodation	670 (1.0)	561 (0.9)	1.404 (1.254,1.57)	604 (0.9)	488 (0.8)	1.482 (1.315, 1.67)	546 (0.8)	432 (0.7)	1.538 (1.355,1.745)
Disorders of lacrimal system	301 (0.5)	232 (0.4)	1.541 (1.298,1.829)	276 (0.4)	200 (0.3)	1.668 (1.39,2.001)	248 (0.4)	170 (0.3)	1.795 (1.476,2.182)
Hordeolum of eyelid	169 (0.3)	191 (0.3)	1.075 (0.874,1.323)	155 (0.2)	180 (0.3)	1.058 (0.853, 1.312)	141 (0.2)	156 (0.2)	1.133 (0.902,1.423)
Blepharitis	92 (0.1)	84 (0.1)	1.32 (0.982,1.775)	83 (0.1)	76 (0.1)	1.337 (0.979, 1.826)	75 (0.1)	71 (0.1)	1.309 (0.946,1.811)
Ocular pain	154 (0.2)	157 (0.2)	1.185 (0.948,1.481)	134 (0.2)	144 (0.2)	1.145 (0.904, 1.449)	120 (0.2)	131 (0.2)	1.144 (0.893,1.466)
Cataract	314 (0.5)	224 (0.3)	1.69 (1.423,2.006)	289 (0.4)	200 (0.3)	1.77 (1.478,2.12)	269 (0.4)	186 (0.3)	1.793 (1.487,2.162)
Disorders of sclera	21 (<0.1)	14 (<0.1)	1.769 (0.899,3.481)	18 (<0.1)	12 (<0.1)	1.807 (0.87,3.755)	15 (<0.1)	11 (<0.1)	1.674 (0.768,3.646)
Glaucoma	146 (0.2)	123 (0.2)	1.419	134 (0.2)	107 (0.2)	1.524	118	102 (0.2)	1.429

			(1.116,1.804)			(1.182,1.966)	(0.2)		(1.096,1.863)
Disorders of vitreous body and globe	178 (0.3)	109 (0.2)	1.964 (1.547,2.493)	163 (0.3)	98 (0.2)	2.031 (1.58,2.61)	154 (0.2)	86 (0.1)	2.216 (1.701,2.886)

EoE, eosinophilic esophagitis; 95% CI, 95% confidence interval; HR, hazard ratio. In the TriNetX analytics platform, to protect patient privacy, any incident case count of 10 or fewer is reported as 10.Propensity score matching was reperformed in each models, with the matched covariates including age, sex, race, body mass index, status of comorbidities (diabetes mellitus, hypertension, hyperlipidemia), status of comedication use (proton pump inhibitors), mental and behavioral disorders due to psychoactive substance use, socioeconomic factors (persons with potential health hazards related to socioeconomic and psychosocial circumstances)

Table S6. Sensitivity analysis of applying different matching covariates

Outcomes	Matching model 1			Matching model 2			Matching model 3		
	EoE cohort (%)[a]	Control cohort (%)	HR (95% CI)[a]	EoE cohort (%)[a]	Control cohort (%)	HR (95% CI)[a]	EoE cohort (%)[a]	Control cohort (%)	HR (95% CI)[a]
Visual disturbance and blindness	971 (1.5)	684 (1.1)	1.568 (1.422,1.73)	971 (1.5)	688 (1.1)	1.578 (1.431,1.741)	806 (1.5)	843 (1.6)	1.166 (1.058,1.284)
Disorders of refraction and accommodation	714 (1.1)	628 (1.0)	1.223 (1.099,1.362)	714 (1.1)	630 (1.0)	1.234 (1.108, 1.373)	516 (1.0)	619 (1.2)	0.996 (0.886,1.120)
Disorders of lacrimal system	320 (0.5)	287 (0.4)	1.201 (1.023,1.408)	320 (0.5)	284 (0.4)	1.229 (1.047, 1.442)	244 (0.5)	241 (0.5)	1.235 (1.033,1.477)
Hordeolum of eyelid	175 (0.3)	202 (0.3)	0.963 (0.786,1.18)	175 (0.3)	204 (0.3)	0.963 (0.787, 1.179)	168 (0.3)	244 (0.5)	0.84 (0.689,1.022)
Blepharitis	98 (0.2)	84 (0.1)	1.278 (0.955,1.711)	98 (0.2)	84 (0.1)	1.292 (0.965, 1.73)	93 (0.2)	93 (0.2)	1.237 (0.927,1.651)
Ocular pain	162 (0.2)	163 (0.3)	1.092 (0.879,1.358)	162 (0.2)	168 (0.3)	1.079 (0.869, 1.34)	132 (0.3)	169 (0.3)	0.969 (0.771,1.217)
Cataract	326 (0.5)	214 (0.3)	1.686 (1.419,2.004)	326 (0.5)	214 (0.3)	1.708 (1.437,2.03)	274 (0.5)	258 (0.5)	1.305 (1.101,1.548)
Disorders of sclera	22 (<0.1)	19 (<0.1)	1.221 (0.661,2.258)	22 (<0.1)	19 (<0.1)	1.252 (0.677,2.314)	17 (<0.1)	14 (<0.1)	1.527 (0.751,3.105)
Glaucoma	157 (0.2)	146 (0.2)	1.18	157 (0.2)	154 (0.2)	1.131	144	132 (0.3)	1.337

			(0.941,1.478)			(0.906,1.414)	(0.3)		(1.055,1.694)
Disorders of vitreous body and globe	187 (0.3)	109 (0.2)	1.896 (1.496,2.402)	187 (0.3)	112 (0.2)	1.865 (1.475,2.358)	149 (0.3)	146 (0.3)	1.258 (1.000,1.582)

EoE, eosinophilic esophagitis; 95% CI, 95% confidence interval; HR, hazard ratio. In the TriNetX analytics platform, to protect patient privacy, any incident case count of 10 or fewer is reported as 10.

Table S7. Sensitivity analysis of excluding for critical confounders

Outcomes	Exclude for asthma			Exclude for allergic rhinitis			Exclude for non-infective enteritis/colitis		
	EoE cohort (%)[a]	Control cohort (%)	HR (95% CI)[a]	EoE cohort (%)[a]	Control cohort (%)	HR (95% CI)[a]	EoE cohort (%)[a]	Control cohort (%)	HR (95% CI)[a]
Visual disturbance and blindness	568 (1.4)	632 (1.5)	1.143 (1.020,1.280)	606 (1.4)	644 (1.5)	1.161 (1.038,1.297)	673 (1.4)	682 (1.4)	1.203 (1.081,1.339)
Disorders of refraction and accommodation	411 (1.0)	484 (1.2)	1.068 (0.936,1.218)	409 (1.0)	498 (1.2)	0.993 (0.871,1.132)	512 (1.1)	602 (1.3)	1.016 (0.903,1.143)
Disorders of lacrimal system	192 (0.5)	169 (0.4)	1.449 (1.177,1.783)	177 (0.4)	184 (0.4)	1.181 (0.961,1.453)	231 (0.5)	213 (0.4)	1.316 (1.092,1.586)
Hordeolum of eyelid	116 (0.3)	169 (0.4)	0.875 (0.691,1.11)	112 (0.3)	158 (0.4)	0.872 (0.684,1.111)	150 (0.3)	197 (0.4)	0.927 (0.749,1.147)
Blepharitis	60 (0.1)	63 (0.2)	1.200 (0.841,1.711)	59 (0.1)	62 (0.1)	1.190 (0.832,1.702)	87 (0.2)	84 (0.2)	1.265 (0.936,1.708)
Ocular pain	113 (0.3)	144 (0.4)	0.992 (0.775,1.271)	108 (0.3)	125 (0.3)	1.067 (0.824,1.381)	119 (0.2)	141 (0.3)	1.033 (0.808,1.319)
Cataract	208 (0.5)	221 (0.5)	1.209 (1.000,1.462)	212 (0.5)	220 (0.5)	1.195 (0.989,1.444)	264 (0.6)	227 (0.5)	1.431 (1.198,1.710)
Disorders of sclera	13 (<0.1)	13 (<0.1)	1.276 (0.590,2.760)	NA			16 (<0.1)	14 (<0.1)	1.411 (0.687,2.899)

Glaucoma	100 (0.2)	110 (0.3)	1.155 (0.880,1.515)	100 (0.2)	105 (0.2)	1.175 (0.893,1.547)	120 (0.3)	102 (0.2)	1.438 (1.103,1.873)
Disorders of vitreous body and globe	121 (0.3)	109 (0.3)	1.433 (1.105,1.858)	112 (0.3)	95 (0.2)	1.472 (1.119,1.936)	131 (0.3)	118 (0.2)	1.376 (1.072,1.766)

EoE, eosinophilic esophagitis; 95% CI, 95% confidence interval; HR, hazard ratio. In the TriNetX analytics platform, to protect patient privacy, any incident case count of 10 or fewer is reported as 10.Propensity score matching was reperformed in each models, with the matched covariates including age, sex, race, body mass index, status of comorbidities (diabetes mellitus, hypertension, hyperlipidemia), status of comedication use (proton pump inhibitors), mental and behavioral disorders due to psychoactive substance use, socioeconomic factors (persons with potential health hazards related to socioeconomic and psychosocial circumstances)
